# Role of Entropy in Colloidal Self-Assembly

**DOI:** 10.3390/e22080877

**Published:** 2020-08-10

**Authors:** Brunno C. Rocha, Sanjib Paul, Harish Vashisth

**Affiliations:** Department of Chemical Engineering, University of New Hampshire, Durham, NH 03824, USA; bc1030@wildcats.unh.edu (B.C.R.); Sanjib.Paul@unh.edu (S.P.)

**Keywords:** entropy, self-assembly, colloids, shape entropy, depletion effects, inverse design

## Abstract

Entropy plays a key role in the self-assembly of colloidal particles. Specifically, in the case of hard particles, which do not interact or overlap with each other during the process of self-assembly, the free energy is minimized due to an increase in the entropy of the system. Understanding the contribution of entropy and engineering it is increasingly becoming central to modern colloidal self-assembly research, because the entropy serves as a guide to design a wide variety of self-assembled structures for many technological and biomedical applications. In this work, we highlight the importance of entropy in different theoretical and experimental self-assembly studies. We discuss the role of shape entropy and depletion interactions in colloidal self-assembly. We also highlight the effect of entropy in the formation of open and closed crystalline structures, as well as describe recent advances in engineering entropy to achieve targeted self-assembled structures.

## 1. Introduction

The second law of thermodynamics leads us to the state function entropy (*S*), a macroscopic property of systems that was discovered by Clausius [1,2]. In an isolated system, entropy increases due to all spontaneous (irreversible) processes and is maximized when the system arrives at equilibrium [3,4,5]. It is often said that the entropy is a measure of molecular order in a system. The higher is the disorder in a system, the higher is the entropy. Therefore, from this perspective, an isolated system undergoing an irreversible process should attain a disordered state to maximize its entropy. However, order or disorder is an imprecise notion that does not quantify the entropy of a system. From a microscopic viewpoint, the entropy of a system is defined by Equation (1) [6,7], where $k_{B}$ is Boltzmann’s constant and *W* is the number of (quantum) states or volume in phase space accessible to the system.

(1) $$S=k_{B}\log W+const.$$

Entropy in a system can be further partitioned into different components: translational, rotational (or orientational), and vibrational. Translational entropy is related to arrangements possible due to translational degrees of freedom, and rotational entropy is associated with the unique ways in which the particles can rotate/orient, while vibrational entropy reflects the vibrational motions of particles [8]. This can be summarized by Equation (2). It is important to note that the translational entropy increases when the excluded volume in the system decreases [9].

(2) $$S=S_{trans}+S_{rot}+S_{vib}$$

Self-assembly, also called ordering, is a well-known phenomenon where individual particles in a system spontaneously organize to form ordered structures [10]. This process usually arises from weak and non-covalent interactions and finds suitable applications in nanotechnology, microelectronics, and photonics [11]. Ordering can be directed by physical characteristics of the particles or via the application of external triggers (e.g., electric and/or magnetic fields, or pressure) [12], or by functionalizing the particle surface [13,14]. For example, an external shear force can cause unidirectional ordering of lamellar bilayers formed by a polymeric surfactant, resulting in the formation of anisotropic hydrogels, which can be used as artificial muscles [15]. In the presence of an external magnetic field, small Fe${}_{3}$O${}_{4}$ nanocubes were observed to self-assemble into different helical superstructures at various concentrations [16].

Like any other spontaneous process, self-assembly is driven by the minimization of the free energy of the system [17]. The change in the free energy of a system at a constant temperature and volume, the Helmholtz free energy (ΔF), is defined by Equation (3), where ΔE is the change in the internal energy of the system and ΔS defines the change in its entropy.

(3) ΔF=ΔE−TΔS

Therefore, a system can lower its free energy either by decreasing the internal energy or increasing the entropy. For ordering phase transitions (e.g., isotropic “disordered” liquids to crystalline “ordered” solids), one can then rationalize that a spontaneous phase transition takes place if the internal energy of the system is sufficiently lowered to outweigh the loss in entropy. While this intuitive interpretation suggests ordering transitions as energy-driven, the opposite has been argued in that the ordering phase transitions are in fact entropy-driven [18]. Therefore, the concept of entropy is central to gain key insights into the mechanisms underlying self-assembly processes.

It is possible to observe the formation of self-assembled structures in biophysical processes, including in DNA assembly [19], protein folding [20], and membrane formation [21], but the mechanisms underlying self-assembly phenomena can also be exploited to produce useful nanostructures [22]. For example, under suitable conditions, colloidal nanospheres self-assemble to form structures that can be utilized as photonic crystals or as models for gaining a better understanding of the crystallization processes [23].

Colloidal particles are small solid particles whose dynamics in suspension are driven by thermal energy fluctuations [24]. They are also known as hard particles since they do not overlap, and the different arrangements of these particles in a system do not cause changes in the internal energy [25]. If the self-assembly occurs under constant internal energy, attractive interactions between the particles emerge solely from the maximization of microstates accessible to the system, which causes an increase in its total entropy [26]. This may sound counterintuitive to the concept of entropy as a measure of visible disorder; however, entropically-driven ordering processes are possible due to a trade-off between the different components of the total entropy (Equation (2)) of the system [25].

For colloids, the application of the definition of entropy in terms of the logarithm of a volume in phase space (often denoted by Ω) is known to result in Gibbs’ paradox and, as a result, the violation of the second law of thermodynamics [27,28,29]. The paradox is rooted in considerations about colloidal particles being distinguishable or not. It has been suggested that the colloidal particles should be considered distinguishable due to each particle having either a different number of atoms or a different arrangement of atoms. Specifically, relating *S* simply to the logarithm of Ω leads to the incorrect conclusion of entropy being non-extensive [27]. However, relating *S* to the logarithm of Ω/N! correctly predicts entropy as an extensive property [28]. Importantly, resolving this paradox for colloids does not necessitate invoking the quantum mechanical notion of indistinguishability that results in the factor of 1/N!.

Early examples of studies related to ordering transitions in colloidal systems include, but are not limited to, Onsager’s hard thin rods [30,31], hard spheres [32,33,34], hard disks [35], and the depletion effect arising in systems composed of colloids and polymers [36]. Other examples of the role of entropy in self-assembly include the formation of binary semiconductor-nanocrystal superlattices [37] and the formation of stable icosahedral clusters by spherical confinement [38]. While studies involving hard spheres provide an important framework for understanding how ordering processes work, not all colloidal particles currently investigated can be well-described under the assumption of a hard sphere. Some colloidal nanocrystals show electrostatic and other non-covalent interactions, as well as the addition of ligands can generate attractive and/or repulsive interactions, which can cause variations in the internal energy of the system [39]. The examples of self-assembly, which are driven by a decrease in the internal energy, include the self-assembly of nano-/micro-particles functionalized with complementary DNA strands [40,41,42,43]. Functionalizing nano-/micro-particles with DNA strands favors the formation of reversible structures during self-assembly (energy driven) and allows tuning of inter-particle interactions over a wide range (0 $k_{B}T$ to 10 $k_{B}T$) by varying the temperature [44,45]. Another example is the self-assembly of gold nanosheets into a thin layer after the addition of ethyl acetate to the aqueous solution, a process driven by the minimization of the free energy due to thermal fluctuations and a lowering of the interfacial tension triggered by the addition of solvent [46]. Therefore, the simulations of such colloidal systems should account for energetic, as well as entropic contributions to provide accurate predictions of self-assembled structures.

Given the undeniable importance of having a good understanding of the role of entropy in self-assembly phenomena, in this work, we primarily highlight and synthesize findings from those published studies that describe entropically-driven ordering transitions and processes. This goal is achieved by discussing the mechanisms underlying self-assembly and highlighting recent developments in the field, including the application of these concepts in molecular simulations that can be utilized to predict various types of self-assembled structures by tuning the particle shape and size, as well as environmental conditions. We also briefly describe the ways in which this knowledge has been applied to optimize experimental procedures.

## 2. Entropic Contributions in Self-Assembly Processes

### 2.1. Effect of Particle Shape in Self-Assembly

The shapes and conformations of particles in a system are driving forces in self-assembly. For example, a concentrated suspension of hard thin rods transitions into a phase where these rods have a preferred orientation and are closer to each other [30]. This ordering happens even though it causes a decrease in the orientational entropy, because the rods will have less available space to undergo changes in their orientation. The reasoning is that the process also causes an increase in the translational entropy of the system, since there will be more volume available per particle, due to which the total entropy of the system increases [47]. The mechanisms underlying the self-assembly phenomena in systems made up of a single type of particle can be described by using a system of hard thin rods as an example.

**Figure 1:**
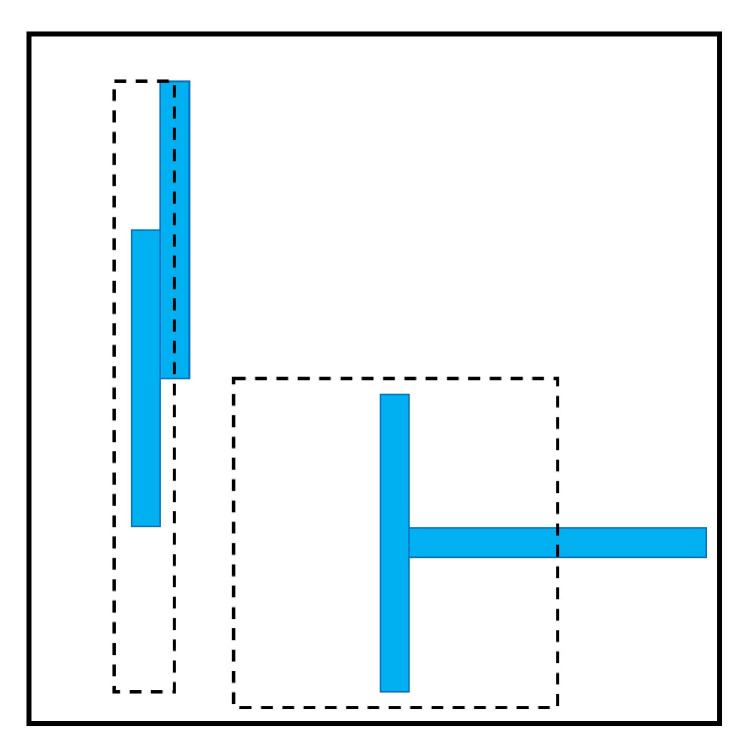
A representation of excluded volume (dashed lines) for different arrangements in a system of hard thin rods. The parallel conformation causes an increase in the overall entropy of the system. (Inspired by Figure 1 in [25]).

Figure 1 shows two rods that are parallel to each other, thus oriented in the same direction, and also shows the same rods in a perpendicular conformation. The dashed lines in both conformations highlight the excluded volume in each conformation of this two-particle system, or the volume that is not accessible to the center of a rod. It is clear that when the rods present a parallel and ordered conformation, there is less excluded volume in the system, which leads to an increase in the translational entropy that is higher than the loss in the orientational entropy. It is important to note that the translational entropy can be gained at the expense of orientational entropy only beyond a certain density [25,31].

**Figure 2:**
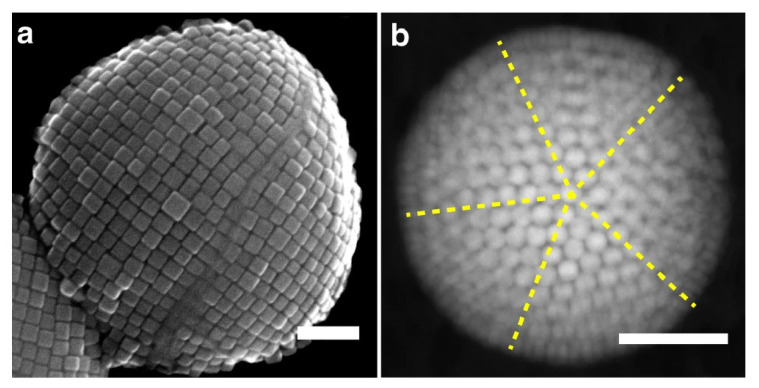
Role of shape in the self-assembly of cubic nanoparticles: (**a**) Simple cubic superstructure formed via self-assembly of sharp cubes. (**b**) Icosahedral-like superstructure formed via self-assembly of round-edge cubes. The size of both scale bars shown is 100 nm. Adapted from [51], with the permission of the Nature Publishing Group under a CC BY 4.0 license.

Based on this effect, it is possible to control the outcome of ordered structures formed by self-assembly processes via tuning of intermolecular interactions through a meticulous design of the particle shape and other environmental conditions (e.g., the temperature of the system) [48]. Tuning of the interactions between colloidal particles, especially through variations in shape and interaction anisotropy, broadens the number of possible structures and provides an extraordinary control over their final conformation [49,50]. For example, the integration of both modeling and experiments has shown that when the shape of sharp cubic nanoparticles is altered to cubes with round edges, their self-assembled structures show an icosahedral-like shape, instead of forming simple cubic superstructures (Figure 2), through an ordering process largely dominated by entropy [51]. For patchy particles, entropic contributions can be tailored by tuning the patch size and geometry [52]. Patches in a chain of patchy particles can also be designed to have their isotropic and anisotropic interactions lead to the formation of useful three-dimensional structures [53,54].

**Figure 3:**
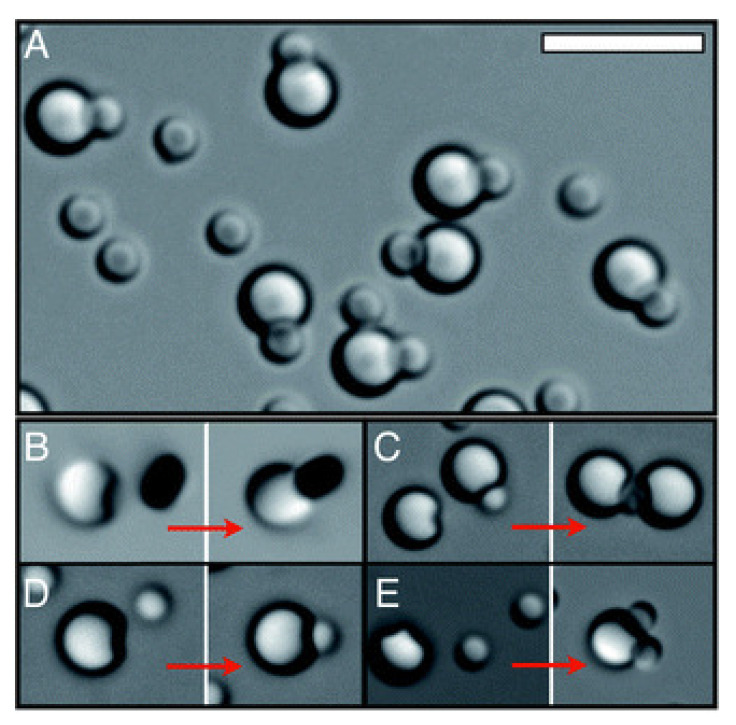
Self-assembly of lock and key pairs: (**A**) Lock-key ratio of 1:2. (**B**–**E**) Binding events between locks and different keys. (**B**) Magnetic hematite ellipsoids. (**C**) Silica spheres. (**D**) Poly(methyl methacrylate) spheres. (**E**) Polystyrene spheres. The size of the scale bar shown is 5 μm. Republished with permission of the Royal Society of Chemistry, from [58]; Permission conveyed through Copyright Clearance Center.

The shape of colloidal particles influences packing, directionality, selectivity, connectivity, and dynamics in self-assembly processes [55]. Experiments have shown that creating cavities in spherical colloidal particles can induce attractive interactions between these modified particles and regular colloidal spheres, forming ordered structures with an effective lock and key recognition mechanism [56]. Since the spheres are free to move inside the cavities, these structures show a high degree of flexibility [57]. Figure 3 shows examples of the self-assembly of lock and key particles [58].

Furthermore, branched colloidal nanoparticles, such as octapod-shaped nanocrystals, can self-assemble to form three-dimensional superlattice structures under proper conditions [59]. Loudet et al. [60] reported differences in the self-assembly behavior of prolate ellipsoids and spheres trapped at an oil-water interface. They observed that the ellipsoid particles self-assembled, forming open structures and chains, while the spheres had random trajectories where they would get close to each other, but then separate again. The distinct behavior shown by the prolate ellipsoid particles stems from entropic effects arising from their unique shape.

**Figure 4:**
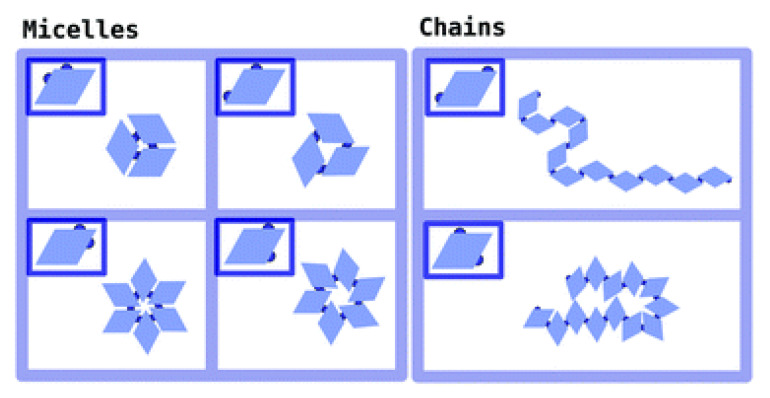
The effect of patch position in the self-assembly of rhombus-shaped particles. Reprinted from [65]. Published by the Royal Society of Chemistry.

Monte Carlo (MC) and Molecular Dynamics (MD) simulations can be used to predict self-assembled structures that maximize the entropy of a system under given conditions [10]. For instance, simulations of colloidal particles grafted with polymer chains of different lengths have predicted the formation of several types of self-assembled structures, including branched cylinders, sheets, and strings, based on the number of monomers in the polymer chain [61]. MD simulations have shown that pear-shaped particles self-assemble, forming gyroid cubic phases [62], and that lobed colloidal particles can form different structures depending on the number of lobes, their positions in the particle, and the temperature of the system [63]. For example, a planar particle with three attractive lobes can self-assemble forming nanotubes or sheets, depending on the thermal energy of the system [63]. However, MC simulations of rhombus-shaped particles decorated with two patches resulted in the formation of chains [64], or micelles [65], depending on the position of the patches in the particle (Figure 4), while neutral nanoparticles with solvophobic chains attached to them were shown to form flat bilayers or spherical aggregates, according to the size and number of chains [66].

Simulations have also shown that hard convex polyhedra self-assemble into different crystalline structures due to the directional entropic forces acting between the particles [67,68]. The role of directional entropic forces during the self-assembly of faceted particles is similar to the role of attractive interactions between the patches in the self-assembly of patchy particles. Therefore, the particles, which experience directional entropic forces during self-assembly, are called entropically patchy particles [69]. The directional entropic force is not an intrinsic property of the particles. It is a collective and statistical property of the self-assembling system, and it increases with an increase in the contact area between two neighboring particles. A series of truncated tetrahedrons, starting from a regular tetrahedron (no truncation) to a regular octahedron (strongly truncated), self-assembles into dodecagonal quasicrystals, diamond crystals, and body-centered cubic crystals depending on the extent of truncation [67]. These truncated tetrahedrons prefer to have a face-to-face alignment in their self-assembled structures to increase the total entropy of the respective systems. The entropy that arises due to the particle shape is often termed as the shape entropy [68]. An amount of entropy, in the case of hard polyhedra, is increased due to the presence of defects or vacancies in the colloidal crystals [70,71]. Smallenburg et al. [70] showed that the vacancies in the crystals of hard cubes provide nearby particles an additional space to move, thereby increasing the entropy of the system.

### 2.2. Self-Assembly Driven by Depletion Effects

Another example of a purely entropically-driven self-assembly process is the ordered arrangement that arises in colloidal systems of large spherical particles when smaller particles (depletants) are added, a process known as the depletion effect [72]. Depletion effects have been shown by MC simulations to be purely entropically-driven [73]. In this type of system, self-assembly of larger particles occurs due to the maximization of the entropy of smaller particles [74].

**Figure 5:**
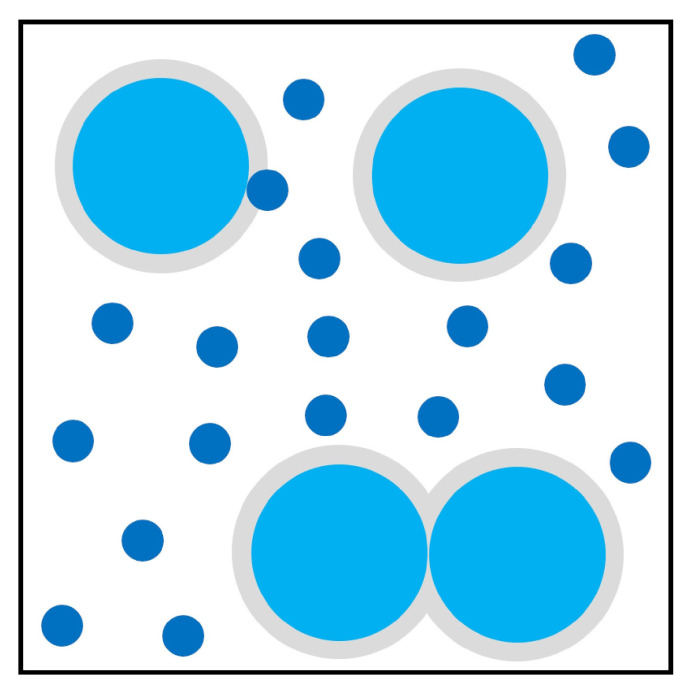
A representation of depletion effects in a colloidal system. Light shaded areas represent excluded volume. When the particles come closer, excluded volume decreases, and the available volume increases. Inspired by Figure 1 in [75].

Figure 5 highlights the ordering transition in this situation by illustrating the reorganization of large colloidal particles when depletants are added to the system. The shaded areas represent the excluded volume of the system, or the volume not accessible to the centers of smaller particles. When these areas overlap, there is an increase in the volume available to smaller particles to move around, thus increasing their entropy. Effectively, the addition of depletants to the system makes larger particles self-assemble into ordered structures by promoting attraction between these particles and the walls.

Depletion effects can be exploited as a potential route to obtain ordered structures in a desired pattern [75], and its effective potential (*u*) is described by Equation (4), where $\rho_{p}$ is the density of the depletant, $k_{B}$ is Boltzmann’s constant, and ΔV is the overlap volume [76].

(4) $$u=-\rho_{p}k_{B}T\Delta V$$

Mathematically, the relationship between the change in available volume and the free energy of the system can be defined by Equations (5) and (6), where Equation (6) is a valid approximation for the partition function (*Q*) when the colloidal system is diluted [77].

(5) $$F=-k_{B}T\;ln\;Q$$

(6) $$Q=V^{N}={\left(V_{0}+\Delta V\right)}^{N}$$

In the latter equation, *N* is the number of particles in the system, $V_{0}$ is the initial accessible volume, and ΔV is the change in the accessible volume due to a change in the arrangement of the system. It describes that an increase in the accessible volume will cause an increase in the partition function *Q*, which leads to a lowering of the free energy of the system, per Equation (5). This further shows that ordering results due to the minimization of the free energy.

**Figure 6:**
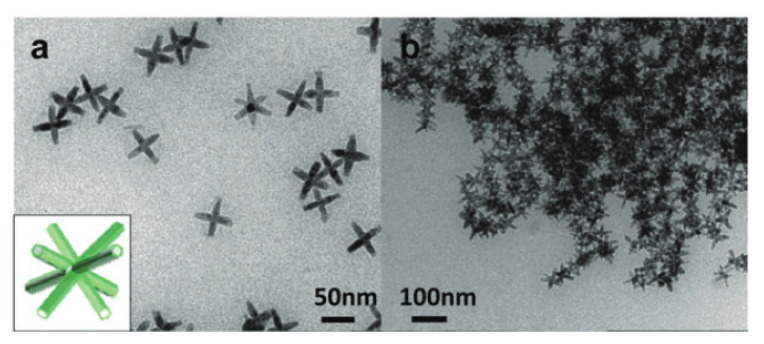
Self-assembly of branched nanocrystals via depletion interactions: (**a**) Non-assembled CdSe/CdS octapods. (**b**) Self-assembled structure induced by the addition of oleic acid. Republished with permission of the Royal Society of Chemistry, from [81]; Permission conveyed through Copyright Clearance Center.

Utilizing depletion interactions is an example of manipulating local entropy to trigger and control self-assembly. The attractive interactions between the larger colloidal particles can be tuned by playing with the concentration and size of the smaller particles [78]. Examples of the use of depletion effects to induce self-assembly include the formation of microsheets from colloidal CdSe/CdS nanorods after the addition of oleic acid and poly(ethylene glycol) methacrylate and shape-selective separation of rods and spheres in a binary mixture [79]. This effect has also been used in the investigation of self-healing systems, where nanoparticles migrate to exposed cracks on the polymer surface, thus improving the durability of these materials [80], and in the self-assembly of branched nanocrystals (Figure 6) [81].

Using depletion interactions, Barry et al. [82] demonstrated the self-assembly of nonamphiphilic membranes from a mixture of model rod (filamentous virus *fd*) and nonadsorbing polymer molecules. Xie et al. [83] studied the self-assembly of faceted photon-upconversion nanorods utilizing the depletion interactions. These nanorods having hexagonal cross-sections and very low aspect ratios were found to self-assemble into nematic and smectic phases with single and multi-axis orientational ordering. Recently, based on the fact that the geometry of particles can affect the overlap volume [84], Kemp et al. [85] showed that the depletion interactions can be controlled by the particle roughness. In a mixture of smooth and rough particles and in the presence of depletants, the smooth particles self-assemble due to higher depletion interactions, and the rough particles remain unassembled.

### 2.3. Role of Entropy in the Formation of Open Lattices

**Figure 7:**
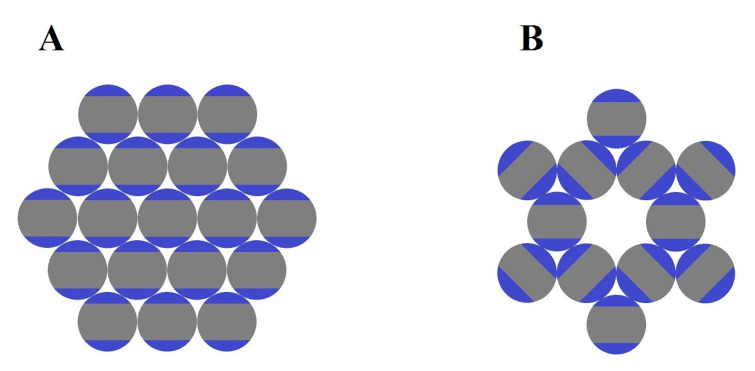
Comparison between self-assembled arrangements formed by triblock Janus particles. (**A**) Hexagonal closed-packed. (**B**) Kagome lattice. Inspired by Figure 1 in [86].

Entropy also plays an important role in the formation of open colloidal lattices [86], which can be defined as a type of arrangement where the structure shows a lower volume fraction, lower coordination number, and an abundance of open spaces between the self-assembled particles [87]. For example, it is observed experimentally that spherical colloids with attractive patches in their poles, known as triblock Janus particles, self-assemble to form Kagome lattice structures, which differ from close-packed arrangements that spheres usually form, such as hexagonal lattices [88]. Particles in Kagome and hexagonal lattices share the same rotational entropy; however, the more crowded nature of close-packed lattices leads to a smaller vibrational entropy. Therefore, Kagome arrangement is favored due to its higher vibrational entropy, which causes a larger minimization in the free energy of the system [89]. Figure 7 shows a comparison between these two types of conformations.

Figure 7A,B show the representation of a hexagonal close-packed lattice and a Kagome lattice arrangement, respectively. The Kagome lattice is a less crowded conformation when compared to the close-packed one, as the attractive patches in triblock Janus particles drive self-assembly to form structures with a higher porosity when compared to the ones obtained from packing of regular spheres [88]. Such porous structures can find suitable applications in photonics [90,91,92] and tissue engineering [93].

MC simulations of systems composed of DNA-coated colloids at lower pressures showed that these particles self-assemble, forming floppy and open crystals instead of compact ones, due to the higher vibrational entropy that these arrangements provide [94]. Moreover, experiments performed by Breen et al. [95] showed that polyhedra of different shapes formed open lattice mesostructures in solutions of potassium bromide. In a recent work, Alberstein et al. [96] studied the self-assembly of a tetrameric protein (a tetramer of L-rhamnulose-1-phosphate aldolase proteins functionalized with four cysteines) into two-dimensional open and closed structures. They revealed that the self-assembled structures prefer to stay in the closed state in the water medium due to the higher solvent entropy.

### 2.4. Relative Entropy and Its Importance in Inverse Design Techniques

Relative entropy ($S_{rel}$) has been introduced as a thermodynamic generating function that quantifies the information lost upon coarse-graining of a system [97]. $S_{rel}$ can be calculated using Equation (7), where $p_{M}\left(i\right)$ and $p_{T}\left(i\right)$ are the probabilities of a given configuration *i* in the ensembles for the model and target structures, respectively. A further examination of Equation (7) shows that when the probabilities of a configuration *i* for both model and target structures are the same, $S_{rel}$ will be equal to zero, meaning that the entropies of both systems are equal, and the Coarse-Grained (CG) model describes the all-atom system accurately.

(7) $$S_{rel}=\sum_{i}p_{T}\left(i\right)\;ln\;\frac{p_{T}\left(i\right)}{p_{M}\left(i\right)}$$

The optimization of CG models is carried out by minimizing $S_{rel}$ between a CG model and a reference all-atom system [98]. The concept of relative entropy has been utilized for the optimization of the parameters of CG models developed for water molecules [99], implicit solvents [100], and peptide systems [101].

Inverse design is a class of computational modeling techniques utilized to discover suitable pair interactions [102,103,104] and particle shapes that can drive self-assembly to a previously designed target structure [105]. These techniques are so-called inverse because their iterative routines start from a target structure and tailor the particles’ interactions or shapes in order to maximize the likelihood of obtaining a predefined configuration [106], as opposed to “regular” simulations that utilize well-defined potentials and shapes to determine the self-assembled structure.

**Figure 8:**
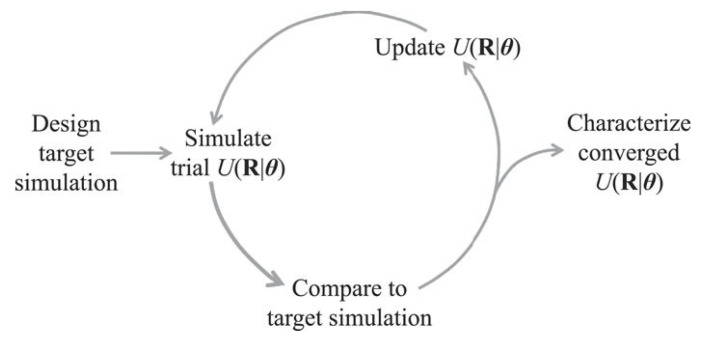
A representation of the iterative process carried out during the inverse design routine. Reprinted from [106], Jadrich et al. *J. Chem. Phys.* 2017, 146, 184103, with the permission of AIP Publishing.

When utilized to determine the optimal pair interactions, inverse design techniques rely on the minimization of the relative entropy ($S_{rel}$) between the model and target structures [107]. Inverse design techniques used to determine pair interactions generally follow the steps described in Figure 8: first, the target structure is designed; second, a system of particles is simulated utilizing an initial guess for the pair potential; third, the model structure obtained from the self-assembly of particles is compared with the target, and the relative entropy is calculated; fourth, pair interactions are revised to minimize the relative entropy, thus maximizing the likelihood of obtaining the target structure [106].

The implementation of this technique for determining pair interactions led to the formation of several two-dimensional target lattices, including rectangular Kagome, triangular honeycomb, and octadecagonal star binary [108], and three-dimensional lattices, like diamond and simple cubic [109].

**Figure 9:**
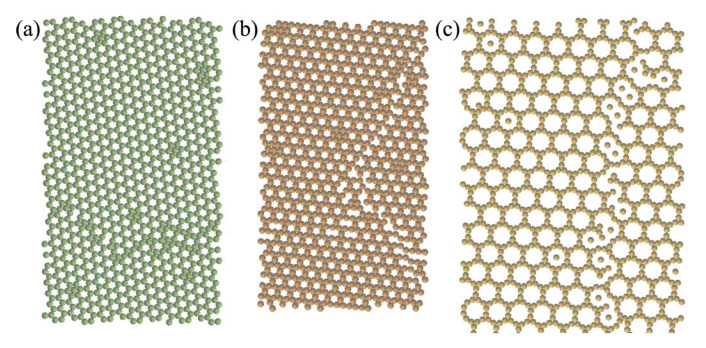
Self-assembled structures obtained using inverse design techniques: (**a**) Honeycomb. (**b**) Kagome. (**c**) Truncated hexagonal lattices. Reprinted from [110], Lindquist et al. *J. Chem. Phys.* 2016, 145, 111101, with the permission of AIP Publishing.

While this technique has been successfully used to obtain pair potentials that favor target structures, some of its limitations include the requirement of specifying relevant competing structures and the fact that a target structure that is formed in the ground state might not be formed when the initial fluid state of the particles is cooled from higher temperatures [110]. To overcome these problems, Lindquist et al. [110] introduced a relative entropy based approach where, in every step of the optimization, an improved pair potential is used to simulate the melting of crystalline structures (obtained from the previous step) at a higher temperature, and then, cooling is performed at a lower temperature to get an improved crystalline structure (closer to the targeted one). This method ensures that a desired structure would be obtained from a disordered fluid state of colloidal particles upon cooling the system. The isotropic pair potentials found using this approach were purely repulsive and generated crystalline structures like honeycomb, Kagome, and truncated hexagonal lattices (Figure 9) [110].

Unlike the usual approach of tuning the pairwise interaction potentials between the particles, tuning the particle shape is a recently developed inverse design method of engineering entropy to produce equilibrium structures via self-assembly [111]. In this method, an extended ensemble is constructed where hundreds of additional coordinates pertinent to the particle shapes are included along with the positional and orientational coordinates to define the phase space. The alchemical MC simulations carried out in these extended ensembles have predicted the optimum particle shapes through the entropy maximization of the targeted structures [111].

## 3. Conclusions

In this work, we discussed the importance of understanding the role of entropy in ordering transitions in colloidal systems. We also described the ways in which control over the shape and size of colloidal particles self-assembles them to form useful structures with suitable properties for applications in technological and biomedical domains. We described the concept of relative entropy and its usefulness for inverse design methods in achieving optimal particle shapes and desired self-assembled structures. However, experimentally synthesizing particles of different shapes at a high precision is challenging. Moreover, tuning entropy is quite infeasible in experiments, but tuning enthalpy or inter-particle interactions is feasible. However, the study of entropically-driven self-assembly processes is a field that has recently gained attention, as computational resources and knowledge evolve to allow simulations of colloidal systems of particles with unique shapes, and under new conditions. The entropy driven self-assembly has been able to generate complex structures because of the meticulous selection of particle shapes, sizes, and concentrations. In any self-assembly process, the use of depletion interactions and shape entropy together may have a greater ability to form structures having more complexity and variety. In those self-assembly processes, where the energy and entropy both play important roles, it is the entropy that often determines the most probable structures among many competing structures. In recent years, theoretical and simulation studies have taken the lead in exploring the effect of entropy in self-assembly processes, and the results obtained from these simulations are being applied to optimize experimental procedures that yield new types of materials with targeted properties, pushing the frontiers of nanotechnology.

## Abbreviations

The following abbreviations are used in this manuscript:

MC	Monte Carlo
MD	Molecular Dynamics
CG	Coarse-Grained
